# Explainable detection of depression in Arabic tweets: identifying clinically meaningful linguistic markers using SHAP

**DOI:** 10.3389/frai.2026.1917139

**Published:** 2026-10-07

**Authors:** Erfan Salehi, Maryam Raissi, Amirhossein Rasouli, Wajdi Zaghouani

**Affiliations:** 1 Department of Clinical Psychology, Faculty of Medicine, Hamedan Branch, Islamic Azad University, Hamedan, Iran; 2 Department of Psychology, Science and Research Branch, Islamic Azad University, Tehran, Iran; 3 Student Research Committee, Department of Clinical Psychology, School of Medicine, Shahid Beheshti University of Medical Sciences, Tehran, Iran; 4 Communication Program, Northwestern University in Qatar, Education City, Doha, Qatar

**Keywords:** Arabic NLP, depression detection, explainable AI, Random Forest, SHAP

## Abstract

**Background:**

Depression detection models for Arabic social-media text often achieve high predictive accuracy but operate as black boxes, limiting their acceptability in clinical and public-health contexts. This study aimed to develop and evaluate an explainable machine learning framework capable of identifying clinically meaningful linguistic markers of depression in Arabic tweets.

**Methods:**

We used the ArabMindGuard dataset (3,079 manually annotated tweets; 21.4% Depressed). Tweets were processed through a standard Arabic NLP pipeline and represented using TF-IDF vectorization (unigrams and bigrams). We compared six classification pipelines (Logistic Regression, Random Forest with three imbalance-handling strategies, SVM, and XGBoost) using 5-fold stratified cross-validation with TF-IDF and resampling strictly inside each fold. The primary model, Random Forest with SMOTE, was selected for its compatibility with exact TreeSHAP computations. To assess generalizability, we externally validated the model on the independent Nassar corpus (*N* = 10,000). TreeSHAP was applied for interpretability, and a systematic error analysis was conducted on all false negatives and false positives.

**Results:**

The RF + SMOTE model achieved an AUC-ROC of 0.80 on the held-out test set and a weighted F1-score of 0.79, with SMOTE improving depressed recall from 0.38 to 0.57 (CV recall = 0.59 ± 0.05). SHAP analysis identified مافيني (lack of energy), نفسيتي (psychological distress), خمول (lethargy), ضيقه (distress) and طاقة مافيني (I have no energy) as the most influential predictors, aligning with DSM-5 symptom domains. External validation on Nassar yielded an AUC of 0.752, with 80% (16/20) of top SHAP features overlapping across datasets. Systematic error analysis of 58 false negatives and 67 false positives revealed that false negatives consistently lacked these prototypical markers, confirming the model's interpretable decision logic.

**Conclusion:**

By combining a transparent classifier with SHAP-based interpretation and cross-dataset validation, this study provides a replicable framework for surfacing clinically meaningful linguistic markers of depression in Arabic text. The approach is intended to support, rather than replace, clinical judgment, and offers a methodological template for transparent digital mental health screening tools in under-resourced languages.

## 1 Introduction

Depressive disorders represent one of the most pressing global health challenges of the modern era, affecting over 300 million individuals worldwide and representing approximately 3.8% of the global population (Aljameel et al., 2026). The World Health Organization has projected that by 2030, depression will become the leading cause of global disability, surpassing cardiovascular disease (Friedrich, 2017). This trajectory carries devastating consequences: a recent comprehensive meta-analysis of 71 countries spanning 122 years of data has corrected the widely underestimated global suicide toll, revealing that more than one million lives are lost annually to suicide once systematic underreporting is accounted for, substantially exceeding the previously cited figure of 727,000 (Daoud et al., 2025). These statistics underscore the urgent need for scalable, accessible, and cost-effective approaches to early detection and intervention.

The mental health crisis is particularly acute in the Arab world, where cultural stigma, limited mental health literacy, and insufficient specialized services converge to create significant barriers to care. In Saudi Arabia specifically, a 2025 systematic review of depression prevalence across Gulf Cooperation Council countries reported that the proportion of Saudi adults experiencing clinically significant depressive symptoms ranges from 2.1% to 77.8%, with a pooled estimate of approximately 37%, substantially exceeding global averages (Alhomaid et al., 2025). The situation among younger demographics is even more alarming: one cross-sectional study of 728 university students in Jeddah found that 81.5% screened positive for depression on the Patient Health Questionnaire-9 (PHQ-9), with no significant difference between medical and non-medical majors (Alzahrani, 2024). Moreover, Aljameel et al. (2026) reported that while 12.7% of the Saudi population is affected by depression, only 1.5% of those diagnosed receive annual treatment, revealing a profound treatment gap exacerbated by societal stigma and limited access to mental health services. Within this context, the development of automated, non-intrusive screening mechanisms that can operate at a population scale becomes not merely a technological aspiration but a public health imperative.

Parallel to this escalating mental health crisis, the digital transformation of social communication has created unprecedented opportunities for population-level mental health surveillance. Social media platforms such as Twitter (now X) have evolved into spaces where individuals spontaneously disclose deeply personal experiences, emotional states, and psychological distress in real time, generating vast repositories of linguistically rich, ecologically valid data. In Saudi Arabia, where approximately 41% of the population actively uses Twitter (Aljameel et al., 2026), the platform offers a uniquely valuable window into the mental health landscape of a society where traditional help-seeking remains constrained by cultural barriers. Research has demonstrated that individuals experiencing depression exhibit distinct linguistic patterns on social media, including increased use of first-person pronouns, negative emotion words, and expressions of social isolation (Reece et al., 2017). These patterns provide opportunities for early detection and intervention, particularly in contexts where traditional mental health services are limited or stigmatized. To address the generalizability gap, recent efforts have introduced additional Arabic depression corpora. Notably, Helmy et al. (2024) developed the Nassar Arabic depression corpus, a publicly available dataset of 10,000 tweets balanced across depressed and non-depressed classes, and demonstrated the effectiveness of sentiment analysis for depression detection in both English and Arabic tweets.

Natural language processing (NLP) and machine learning (ML) techniques have been increasingly applied to the automated detection of depression from social media text. However, Arabic NLP presents unique and substantial challenges that distinguish it from work conducted on English and other well-resourced languages. As a Semitic language with complex morphological structures, Arabic exhibits extensive prefixation, suffixation, and infixation that generate thousands of possible word forms from a single trilateral root (Habash, 2010). Furthermore, spoken Arabic varies dramatically across regions, with substantial differences in vocabulary, grammar, and expression patterns between Gulf, Levantine, Egyptian, and Maghrebi dialects, differences that significantly complicate automated text analysis (Aljameel et al., 2026). The Arabic script additionally lacks short vowels in most written forms, creating inherent ambiguity in word interpretation that requires sophisticated disambiguation techniques. These linguistic complexities necessitate specialized approaches for Arabic mental health text analysis, making research in this domain particularly valuable for advancing both NLP methodology and public health outcomes in the Arab world.

Despite growing scholarly interest in automated mental health detection, several critical gaps persist in Arabic-language research. A recent comprehensive scoping review by Alasmari (2025) systematically examined the state of Arabic NLP for mental health and identified that while models such as AraBERT and MARBERT have demonstrated accuracy rates as high as 99.3% on specific tasks, the overwhelming majority of existing studies treat their models as opaque decision-making systems that provide no insight into which linguistic features actually drive their predictions. This black-box characteristic constitutes a fundamental barrier to clinical translation, as mental health practitioners are understandably reluctant to base high-stakes decisions about patient care on predictions whose provenance cannot be understood, scrutinized, or challenged (Lundberg and Lee, 2017).

In response to this interpretability crisis, the field of explainable artificial intelligence (XAI) has developed a range of *post-hoc* methods designed to illuminate the internal logic of complex models. Among these techniques, SHapley Additive exPlanations (SHAP), a theoretically grounded explanation framework derived from cooperative game theory, has emerged as the dominant approach for model interpretability across biomedical and clinical applications (Lundberg et al., 2020). A recent systematic review and meta-analysis of explainable machine learning for clinical depression detection by Trelles et al. (2025) found that SHAP was the primary interpretability method employed in 70% of the 20 studies analyzed, and that studies combining SHAP with complementary techniques such as LIME demonstrated superior methodological rigor. In the context of Arabic mental health detection, SHAP-based interpretability has been applied to suicidal ideation detection from social media text (Hasan et al., 2025) but its application to Arabic-language depression detection remains virtually unexplored, constituting the primary research gap that the present study addresses.

Recent efforts have begun to address the intersection of Arabic NLP and mental health. Early efforts in this domain include the work of Almouzini and Alageel (2019), who conducted one of the first studies to detect depressed users from Arabic Twitter data in the Gulf region, establishing an early benchmark for Arabic mental health NLP. Hassib et al. (2022) introduced AraDepSu, a dataset for detecting depression and suicidal ideation in Arabic tweets, and demonstrated the effectiveness of transformer-based models. Abdulsalam et al. (2024) developed an Arabic suicidality dataset and reported that AraBERT achieved 91% accuracy for identifying suicidal ideation. Rabie et al. (2025) proposed a recognition model for major depressive disorder from Arabic user-generated content, achieving competitive accuracy with Multilingual BERT. Similarly, Al-Musallam and Al-Abdullatif (2022) applied machine learning classifiers to detect depressive tweets from Saudi Arabian users, further confirming the feasibility of automated screening in the Saudi context. El-Ramly et al. (2021) introduced CairoDep, a BERT-based approach for detecting depression in Arabic social media posts, demonstrating the potential of transformer models for Arabic mental health text classification. Helmy et al. (2024) introduced the Nassar Arabic depression corpus, a publicly available dataset of 10,000 tweets balanced across depressed and non-depressed classes, and applied sentiment analysis for depression detection in both English and Arabic tweets. Most recently, Zaghouani et al. (2026) introduced AraStress, the first large-scale Arabic corpus for psychological stress research. However, none of these studies incorporated explainability techniques; they remain black-box classifiers that offer no insight into the linguistic features driving their predictions (Hassib et al., 2022; Abdulsalam et al., 2024).

In parallel, explainable AI approaches have been applied to mental health detection in other languages, with SHAP emerging as the dominant method for identifying clinically relevant predictors from both survey data and social media text (Malhotra and Jindal, 2023; Tanaka et al., 2025). The application of such methods to Arabic depression detection remains virtually unexplored, and no study to date has combined SHAP-based interpretability with systematic error analysis and cross-dataset validation in the Arabic social-media context- a gap that the present work directly addresses.

The present study fills this critical gap by developing an explainable machine learning framework for depression detection from Arabic tweets, combining a Random Forest classifier with comprehensive SHAP-based interpretability analysis. Random Forest was selected as the primary model because its tree-based architecture enables exact TreeSHAP computations, providing theoretically guaranteed, locally accurate Shapley value attributions without approximation errors (Lundberg et al., 2020). The utility of Random Forest in this domain has been previously demonstrated by AlSagri and Ykhlef (2020), who employed content and activity features for depression detection on Twitter.

To provide a comprehensive benchmark, we compare six classification pipelines (including Logistic Regression, SVM, XGBoost, and multiple imbalance-handling strategies) using 5-fold stratified cross-validation with TF-IDF and resampling applied strictly inside each fold to prevent data leakage. To assess generalizability beyond the Saudi-specific AMG dataset, we externally validate the model on the independent Nassar corpus (Helmy et al., 2024) and evaluate the stability of SHAP-identified linguistic markers across datasets. Additionally, we conduct a systematic error analysis by examining all false negatives and false positives, quantifying the prevalence of top SHAP markers in each error group to diagnose the model's primary failure modes. Grounded in this overarching aim, we address three specific research questions:

RQ1: To what extent can a Random Forest classifier (trained on TF-IDF features and augmented with SMOTE) provide a sufficiently reliable basis for SHAP-based interpretability, as assessed through standard classification metrics?
RQ2: Which Arabic words and expressions emerge as the most globally influential features driving the model's predictions, as quantified by mean absolute SHAP values, and do these features correspond to clinically recognized markers of depressive language?
RQ3: How do these features behave at the level of individual predictions (as revealed through local SHAP waterfall plots) and across the range of their TF-IDF values, and what additional clinical insights do such fine-grained explanations yield?

## 2 Method

### 2.1 Dataset

The present study utilizes the ArabMindGuard (AMG) dataset, a publicly available collection of Arabic tweets specifically compiled and manually annotated for depression detection research. This dataset was first introduced by Aljameel et al. (2026) and originally comprises 3,083 tweets collected from users in Saudi Arabia. Each tweet was independently labeled by expert human annotators as either Depressed (1) or non-depressed (0) through a rigorous annotation process. Following the data cleaning procedures described below, the final dataset consisted of 3,079 tweets, with a substantial class imbalance: 659 tweets (21.4%) were labeled as Depressed, while the remaining 2,420 (78.6%) were non-depressed. The dataset is openly accessible and has been previously employed as a benchmark for comparing various machine learning classifiers in the domain of Arabic mental health text classification.

The decision to adopt the AMG dataset was driven by two primary considerations. First, the availability of expertly annotated Arabic-language corpora for mental health research remains critically limited, and the AMG dataset addresses this gap directly (Hassib et al., 2022; Alaskar and Ykhlef, 2022). Second, using this dataset allows the present work to directly align with and extend the baseline results reported by Aljameel et al., thereby embedding the current study within an established methodological context while advancing it through the integration of explainable artificial intelligence techniques. To assess generalizability, we additionally evaluated the model on the independent Nassar Arabic depression corpus (Helmy et al., 2024) which comprises 10,000 publicly available tweets balanced across depressed and non-depressed classes.

### 2.2 Data preprocessing

All tweets were subjected to a standardized Arabic natural language processing pipeline that closely follows established practices in the field of Arabic mental health text analysis (Abdulsalam et al., 2024; Elmajali and Ahmad, 2024). The first step involved the removal of all non-Arabic and platform-specific elements. Specifically, URLs, user mentions (e.g., @username), and hashtags (e.g., #topic) were stripped using regular expressions, as these tokens predominantly convey metadata or platform affordances rather than psychological content. Following this, all Arabic diacritical marks [including Fatha, Kasra, Damma, and the elongation character Tatweel (kashida)] were eliminated to normalize orthographic variation and reduce feature sparsity.

The tweets were then filtered to retain only Arabic-script characters (Unicode range \u0600–\u06FF) and whitespace; punctuation marks, Latin characters, digits, and emojis were discarded. Subsequently, Arabic stop words from the NLTK library were removed, and this list was augmented with a custom set of high-frequency function words and religious expressions such as ربنا, اللهم, الله, and والله, which appear pervasively in Arabic discourse but may introduce noise into feature extraction when the goal is to identify psychologically salient vocabulary (Maghraby et al., 2022). Finally, any tweet that contained fewer than two tokens after cleaning was excluded from the dataset to ensure that each instance retained sufficient linguistic content for meaningful vectorization. The cleaned dataset was then split into training (80%) and test (20%) partitions using stratified random sampling, thereby preserving the original class distribution in both subsets.

### 2.3 Feature extraction

To convert the preprocessed Arabic text into a numerical representation suitable for machine learning, the study employed the Term Frequency–Inverse Document Frequency (TF-IDF) weighting scheme. TF-IDF was chosen for two interrelated reasons. First, it constitutes the most widely adopted feature representation method in traditional machine learning approaches to Arabic depression detection (Aljameel et al., 2026), thus ensuring direct comparability with prior benchmark results. Second, in contrast to black-box dense embeddings generated by deep learning models, TF-IDF features operate at the token level and are therefore inherently interpretable, a property that is essential for the subsequent SHAP-based explainability analysis.

The vectorizer was configured with a maximum of 1,500 features in order to balance computational efficiency with representational richness. Both unigrams and bigrams (n-gram range 1–2) were included because previous research has demonstrated that depressive language in Arabic frequently manifests through multi-word expressions such as طاقة مافيني (I have no energy) and لله الحمد (praise be to God), which carry distinct psychological connotations that cannot be captured by individual words alone (Aljameel et al., 2026; Musleh et al., 2022). To filter out noisy, infrequent terms, a minimum document frequency of 3 was imposed, while a maximum document frequency threshold of 0.8 was applied to eliminate corpus-specific stop words that were not captured by the initial stop word list. The resulting 1,500 features consist of specific Arabic tokens (words and bigrams) drawn from the training corpus, with feature values representing the normalized TF-IDF weight of each token within each tweet.

### 2.4 Model training

To build a comprehensive benchmark and ensure a fair comparison, we implemented six classification pipelines. A Logistic Regression (LR) model with class weighting (class_weight = “balanced”) served as an interpretable baseline. For Random Forest (RF), we compared three imbalance-handling strategies: class weighting alone (RF + ClassWeight), Random Over-Sampling (RF + ROS), and the Synthetic Minority Over-sampling Technique (RF + SMOTE). We additionally included a Support Vector Machine with a linear kernel and SMOTE (SVM + SMOTE), and an XGBoost classifier with SMOTE (XGB + SMOTE). The RF + SMOTE pipeline was selected as the primary model because its tree-based architecture enables exact TreeSHAP computations for interpretability, a central requirement of this study.

For all resampling methods, the augmentation was applied exclusively to the training partition after TF-IDF vectorization, generating synthetic samples for the minority (Depressed) class to achieve perfect balance, while leaving the test set untouched. RF and XGBoost were configured with 100 estimators; SVM used a linear kernel with max_iter = 3,000; and class_weight = “balanced” was applied where applicable (LR, RF + ClassWeight, and SVM). All models used a fixed random seed (random_state = 42) for reproducibility.

To assess the stability of all pipelines and prevent data leakage, we performed 5-fold stratified cross-validation (CV) with TF-IDF fitting and resampling applied strictly inside each fold. The mean and standard deviation of depressed-class recall, F1-score, and ROC AUC were recorded across folds. For the primary RF + SMOTE model, we additionally performed threshold tuning using cross-validated predicted probabilities to optimize the F1-score on the minority class.

### 2.5 Explainability analysis with SHAP

For the present study, SHAP values were computed using the TreeSHAP algorithm, specifically designed for tree-based models, on the full test set of 616 instances. All SHAP values were computed in probability space; therefore, each feature contribution reflects an additive change in the predicted probability of the Depressed class. Unlike preliminary experiments that used a 100-sample subset, the present analysis operates on all available test data to ensure that global feature rankings and local explanations reflect the complete test distribution. To assess the generalizability of the learned linguistic markers, SHAP analysis was additionally applied to the independent Nassar corpus (*N* = 10,000), enabling a direct comparison of the top influential features across datasets.

The SHAP analysis was conducted at three complementary levels of granularity. Global interpretability was examined by computing the mean absolute SHAP value for each feature across all 616 instances, which yielded a ranked list of the most globally influential words. These global results were visualized using a bar plot and a summary beeswarm plot (see Section 3). This global importance approach follows the procedures adopted in previous explainable mental health detection studies (Malhotra and Jindal, 2023; Tanaka et al., 2025). Local interpretability was then explored by generating SHAP waterfall plots for three representative test instances (a correctly classified Depressed tweet, a correctly classified non-depressed tweet, and a false negative), which together illustrate the model's decision logic across different prediction outcomes (see Section 3).

Finally, feature behavior was analyzed through SHAP dependence plots for the four most important words, which reveal the relationship between the feature values (TF-IDF weights) and their corresponding SHAP contributions (see Section 3). Such plots make it possible to determine whether the effect of a given word is monotonic, threshold-dependent, or highly context-sensitive, providing deeper insight into the nature of the learned associations (Lundberg et al., 2020).

### 2.6 Evaluation metrics

Model performance was assessed using a comprehensive set of standard classification metrics. Per-class precision, recall, and F1-score were computed alongside macro and weighted averages to provide a nuanced picture of performance across both the majority and minority classes. In addition, the Area Under the Receiver Operating Characteristic curve (AUC-ROC) and the Precision-Recall curve with Average Precision (AP) were reported to capture the model's overall discriminative ability, particularly under conditions of class imbalance. A confusion matrix was generated for the primary RF + SMOTE model to enable detailed error analysis. To ensure robust and unbiased evaluation, 5-fold stratified cross-validation was conducted for all six pipelines, with TF-IDF and resampling applied strictly inside each fold, reporting the mean and standard deviation of recall, F1, and AUC. This multi-metric evaluation framework follows the enhanced standards advocated by Aljameel et al. (2026) and ensures a thorough and transparent performance assessment.

Additionally, a systematic error analysis was performed by identifying all false negatives (FN: depressed tweets misclassified as non-depressed) and false positives (FP: non-depressed tweets misclassified as depressed) in the held-out test set. For each error group, the prevalence of the top 10 SHAP-identified depressive markers was quantified to diagnose the model's primary failure modes.

### 2.7 Ethical considerations

This study utilized the publicly available, anonymized ArabMindGuard dataset. No personally identifiable information was collected or processed. Nevertheless, automated depression detection from social media text raises important ethical concerns. Users may not have explicitly consented to the use of their posts for mental health research. Furthermore, algorithmic predictions based on linguistic markers carry risks of stigmatization and misinterpretation. Specifically, false positives could cause unnecessary distress, while false negatives such as those identified in our error analysis could represent missed opportunities for support. Any potential real-world application of this framework should therefore be limited to research and screening support, with human oversight and in compliance with relevant data protection regulations.

## 3 Results

The dataset consisted of 3,079 tweets, with a notable class imbalance: 2,420 (78.6%) labeled as non-depressed and 659 (21.4%) as Depressed (Table 1). Interestingly, depressed tweets tended to be shorter on average (mean characters = 79.8, SD = 72.2) compared to non-depressed tweets (mean = 101.8, SD = 78.1). A preliminary frequency analysis of the most common terms (after stop word removal) further revealed distinct lexical profiles for each class. Words such as نفسيتي (my psyche), مافيني (I don't have energy), تعبانه (tired), تبلد (numbness), and خمول (lethargy) were among the most frequent terms in the depressed class, while more general conversational words like الحياة (life), اليوم (today), and الناس (people) were prevalent in the non-depressed class.

**Table 1:** Descriptive statistics of the cleaned dataset, overall and by depression class.

Statistic	Overall	Non-depressed	Depressed
Number of tweets	3,079	2,420	659
Percentage	100%	78.6%	21.4%
Avg. characters (SD)	97.1 (77.4)	101.8 (78.1)	79.8 (72.2)
Avg. words (SD)	18.3 (14.5)	19.0 (14.4)	15.6 (14.2)

The preliminary lexical frequency analysis mentioned in the text (e.g., frequent words in each class) is based on token counts after stop word removal and is not shown in this table.

### 3.1 Model performance comparison

We evaluated six classification pipelines using 5-fold stratified cross-validation with TF-IDF and resampling applied strictly inside each fold. Table 2 summarizes the performance of all models on both cross-validation and the held-out test set (*N* = 616). Figure 1 presents the confusion matrix for the primary RF + SMOTE model, and Figure 2 shows the corresponding ROC and Precision-Recall curves.

**Table 2:** Performance comparison of logistic regression and Random Forest classifiers.

Model	Evaluation	Accuracy	Recall (Dep)	F1 (Dep)	AUC-ROC
Logistic regression *(baseline, class_weight = “balanced”)*	**5-fold CV**	0.796 ± 0.013	0.681 ± 0.046	0.588 ± 0.024	0.849 ± 0.016
	Test set	0.77	0.62	0.54	
RF + ClassWeight	**5-fold CV**	0.820 ± 0.008	0.387 ± 0.027	0.479 ± 0.026	0.819 ± 0.021
RF + ROS	**5-fold CV**	0.819 ± 0.014	0.522 ± 0.037	0.553 ± 0.035	0.815 ± 0.023
RF + SMOTE *(primary model)*	**5-fold CV**	0.812 ± 0.013	0.526 ± 0.059	0.544 ± 0.042	0.813 ± 0.021
	Test set (thr = 0.5)	**0.80**	0.56	0.54	**0.80**
	Test set (thr = 0.45)	0.77	0.61	0.53	
SVM + SMOTE *(linear kernel)*	**5-fold CV**	0.791 ± 0.005	**0.672** **±0.041**	**0.578** **±0.013**	**0.830** **±0.010**
XGBoost + SMOTE	**5-fold CV**	0.803 ± 0.018	0.495 ± 0.041	0.518 ± 0.039	0.815 ± 0.025
RF + SMOTE *(external validation)*	**Nassar corpus** *(N = 10,000)*	**0.61**	**0.26**	**0.40**	**0.752**

CV results are mean ± std over 5 stratified folds, with TF-IDF and resampling applied strictly inside each fold. Test set (*N* = 616) uses default threshold (0.5) unless stated. Threshold tuning for RF + SMOTE (0.45) was performed using CV probabilities. External validation on Nassar corpus (*N* = 10,000) reports the performance of the AMG-trained RF + SMOTE model without fine-tuning. Bold values indicate the best performance for each metric among all models.

**Figure 1:**
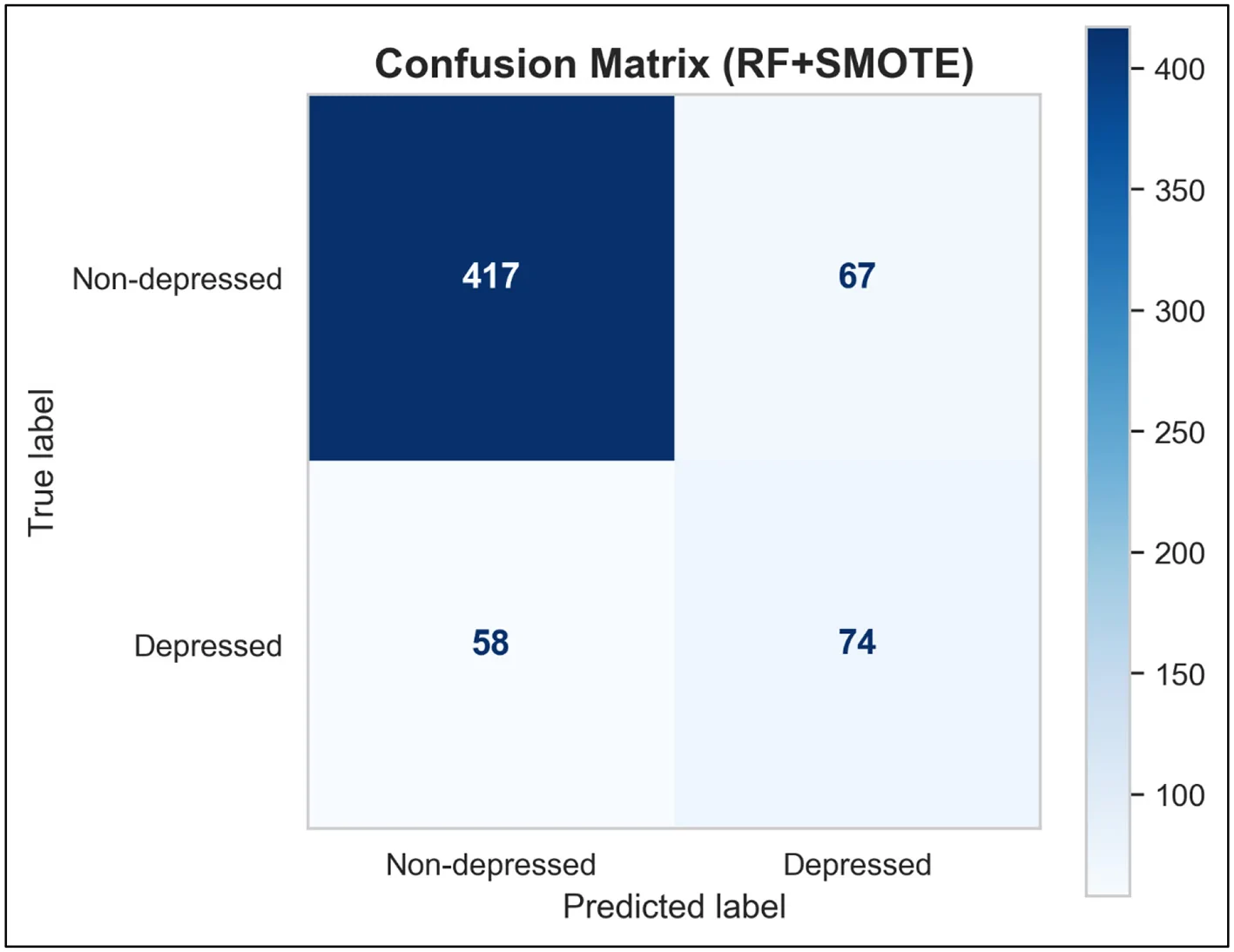
Confusion matrix of the Random Forest classifier trained with SMOTE, evaluated on the test set (*N* = 616).

**Figure 2:**
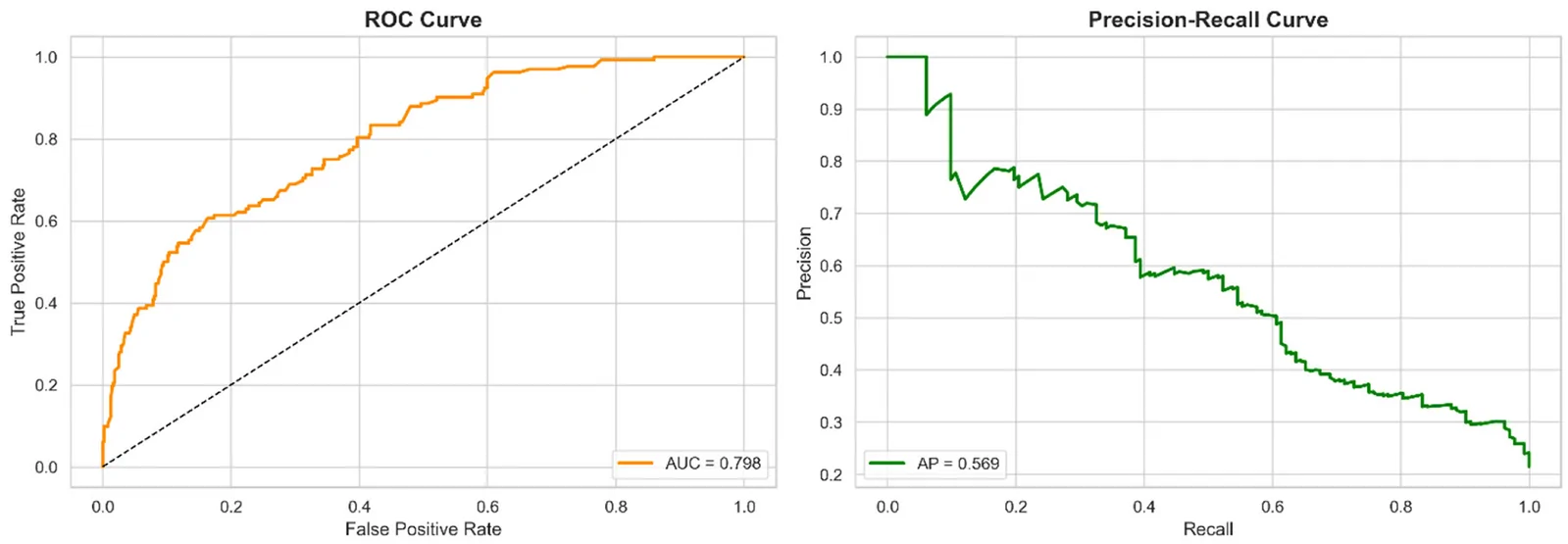
Receiver operating characteristic (ROC) curve (AUC = 0.80) and precision-recall curve (AP = 0.57) for the Random Forest + SMOTE model.

Among the Random Forest variants, class weighting alone yielded a depressed recall of 0.387 ± 0.027, while ROS (recall = 0.522 ± 0.037) and SMOTE (recall = 0.526 ± 0.059) substantially improved minority-class detection. SVM + SMOTE achieved the highest depressed recall (0.672 ± 0.041) and F1 (0.578 ± 0.013), followed by Logistic Regression (recall = 0.681 ± 0.046, F1 = 0.588 ± 0.024). XGBoost + SMOTE yielded intermediate performance (recall = 0.495 ± 0.041, F1 = 0.518 ± 0.039).

On the held-out test set, RF + SMOTE with the default threshold (0.5) achieved an accuracy of 0.80, depressed recall of 0.56, F1 of 0.54, and AUC of 0.80. Compared to the initial RF trained without resampling (recall = 0.38, F1 = 0.47, obtained in preliminary experiments), SMOTE substantially narrowed the performance gap between classes. Threshold tuning using cross-validated probabilities identified an optimal threshold of 0.45, which improved depressed recall to 0.61 at the cost of precision (F1 = 0.53).

On the independent Nassar corpus (*N* = 10,000, 50% depressed), the AMG-trained RF + SMOTE model achieved an AUC of 0.752, with depressed recall of 0.26 and F1 of 0.40 (Table 2). SHAP analysis on the Nassar corpus revealed that 16 out of 20 (80%) of the most influential features overlapped with those identified in AMG (Table 3).

**Table 3:** Stability of SHAP-identified features across AMG and Nassar corpora.

Rank	AMG (primary dataset)	Nassar (external corpus)	Overlap
1	مافيني	اكتئاب	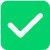 Yes
2	نفسيتي	مافيني	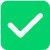 Yes
3	خمول	نفسيتي	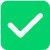 Yes
4	ضيقه	طاقهمافيني	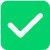 Yes
5	طاقهمافيني	ضيقه	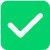 Yes
6	تعبانه	خمول	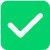 Yes
7	تبلد	تعبانه	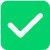 Yes
8	مالي	فيني	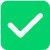 Yes
9	فيني	يارب	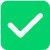 Yes
10	ابي	عانيت	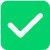 Yes
11	عانيت	احس	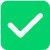 Yes
12	اكتئاب	سعيدة	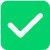 Yes
13	تعاسه	ابي	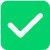 Yes
14	انخذلت	تبلد	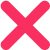 No
15	اليوم	سعادة	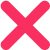 No
16	شرايكم	نفسي	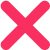 No
17	نفسي	مالي	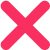 Yes
18	طاقه	طاقه	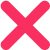 Yes
19	مو	ابغى	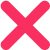 No
20	يارب	تعاسه	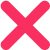 Yes

Overlap = 16/20 (80%). Shaded rows indicate non-overlapping features.

### 3.2 SHAP-based explainability analysis

SHAP analysis revealed three complementary levels of insight into the model's predictions: global feature importance, feature-effect dependence, and local instance-level explanations. The following subsections report these findings.

### 3.3 Global feature importance

Figure 3 displays two complementary visualizations of global feature importance. The left panel is a bar plot of mean absolute SHAP values for the 15 most influential features across all 616 test instances; the right panel is a beeswarm summary plot showing the full distribution of SHAP values for those same features, with point color encoding the TF-IDF value.

**Figure 3:**
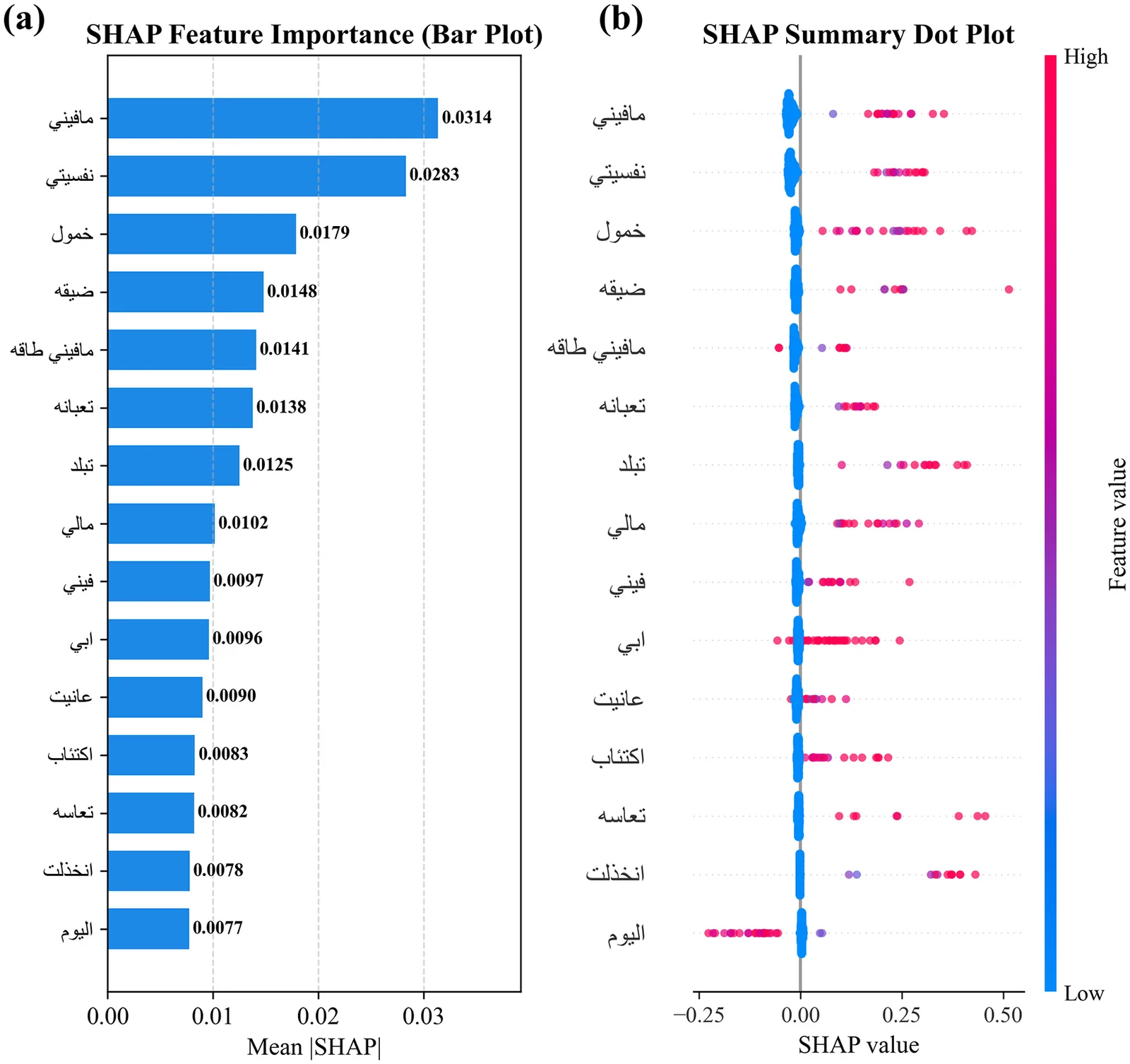
SHAP global feature importance. **(a)** Bar plot of mean |SHAP| for the top 15 features. **(b)** Beeswarm summary plot.

The five features with the highest mean absolute SHAP values were مافيني (0.0314), نفسيتي (0.0288), خمول (0.0179), ضيقه (0.0148), and مافيني طاقه (0.0141). In the beeswarm plot, instances with high TF-IDF values (red points) were predominantly located to the right of the zero line, whereas instances with low TF-IDF values (blue points) were clustered near zero or in the negative range. This pattern was consistent across the top 15 features: higher TF-IDF values were associated with larger positive SHAP contributions, while lower values corresponded to contributions near zero or negative.

### 3.4 Feature-effect dependence

Figure 4 presents SHAP dependence plots for the four most influential features, illustrating the relationship between each feature's TF-IDF value and its corresponding SHAP contribution.

**Figure 4:**
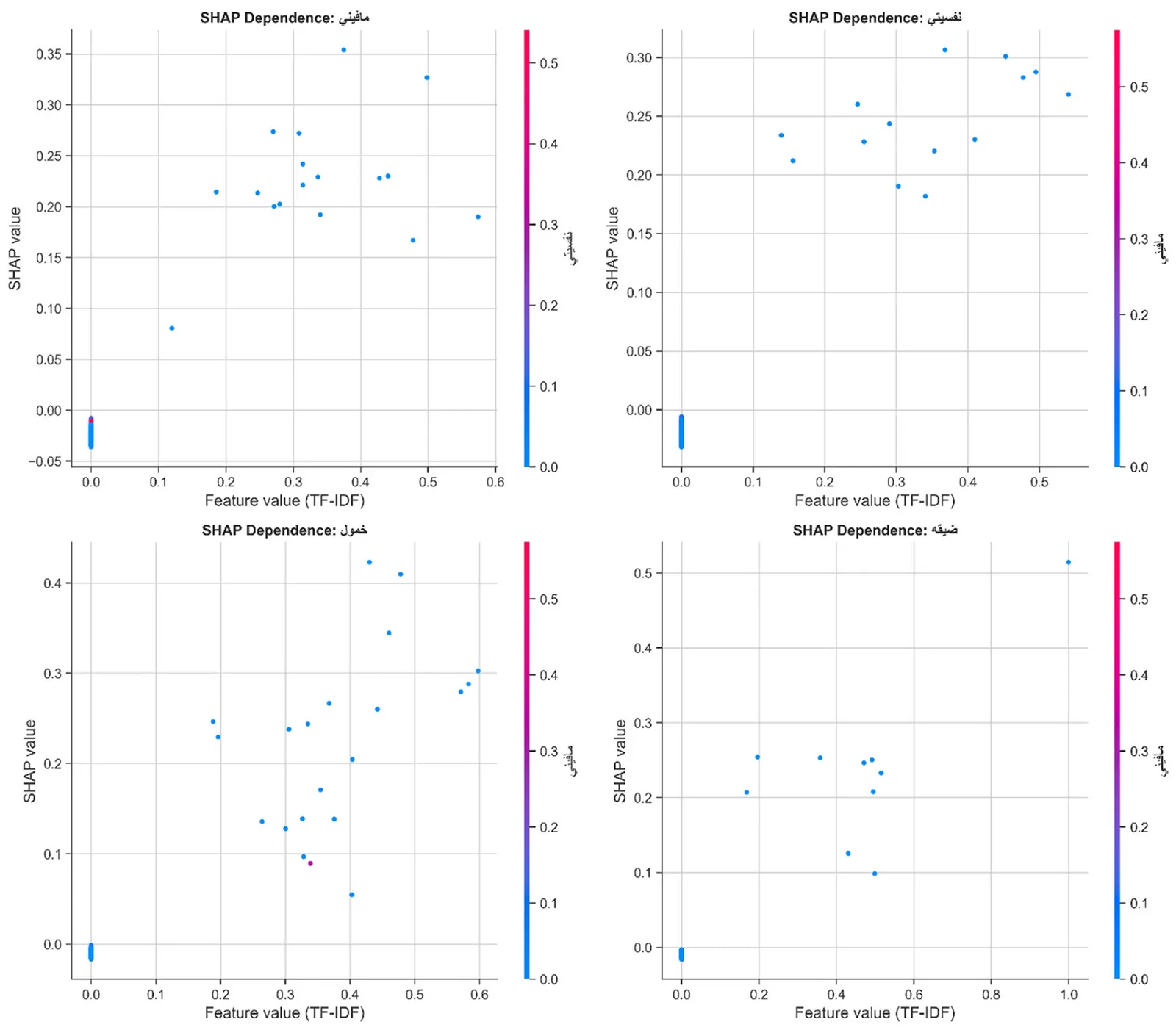
SHAP dependence plots for the four most influential features.

For مافيني, SHAP values increased from approximately −0.01 at zero TF-IDF to a plateau above + 0.05 when a non-zero value was observed, reaching a maximum of approximately +0.12 at the highest frequencies. A similar positive trend was observed for نفسيتي, though with a slightly broader spread of SHAP values at mid-range TF-IDF values (0.1–0.3). For ضيقه, a positive SHAP shift (approximately +0.02) was observed even at minimal TF-IDF values (≈0.05). No instances with high TF-IDF values for these features fell into the negative SHAP region.

### 3.5 Local instance-level explanations

Figure 5 presents SHAP waterfall plots for three representative test instances. In each plot, the base value E[f**(X)**] = 0.545 represents the model's expected output over the background dataset; red bars increase the prediction toward the Depressed class, blue bars decrease it; and the final value f(x) is the predicted probability of the Depressed class.

**Figure 5:**
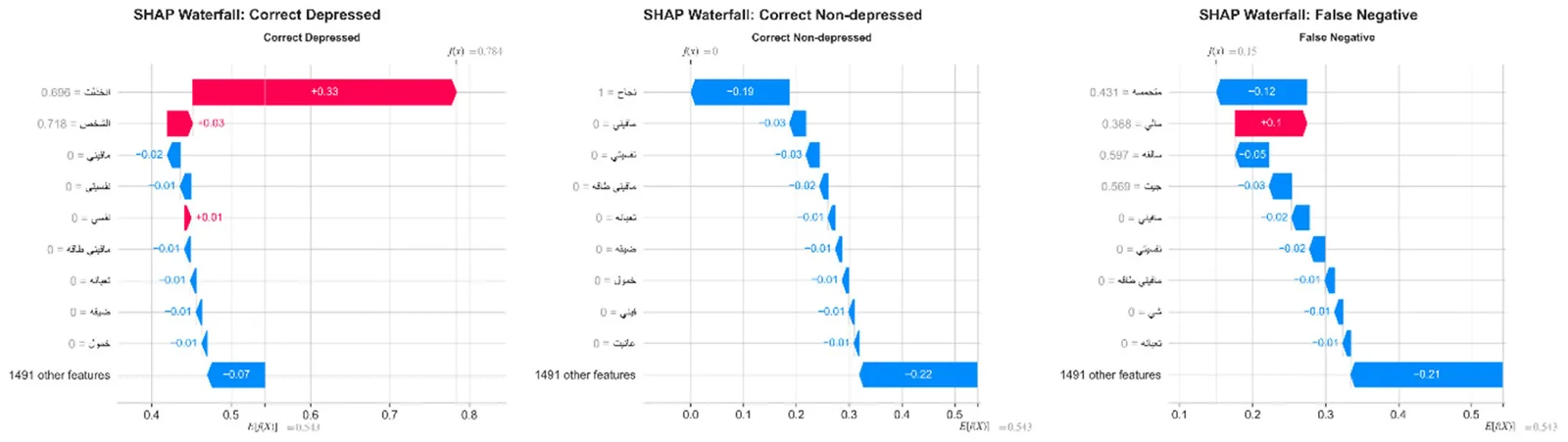
SHAP waterfall plots for three representative test instances.

In the correctly classified depressed tweet (left panel), the word انخذلت contributed a positive SHAP value of +0.33, raising the final probability to 0.784, while a negative contribution from سعادة (−0.05) was outweighed. In the correctly classified non-depressed tweet (middle panel), the word نجاح contributed −0.19, resulting in a final probability of 0.00; the prototypical depressive markers (خمول, نفسيتي, مافيني) were absent. In the false negative case (right panel), the word متحصله contributed −0.12, and the only positive contribution was from مالي (+0.06), yielding a final probability of 0.15; the high-impact depressive markers identified in the global analysis (خمول, انخذلت, مافيني) were not present.

### 3.6 Systematic error analysis

To systematically investigate the model's failure modes, we examined all false negatives (FN: depressed tweets misclassified as non-depressed) and false positives (FP: non-depressed tweets misclassified as depressed) in the test set. Among 616 test instances, there were 58 FNs and 67 FPs.

Table 4 reports the prevalence of the top 10 SHAP-identified depressive markers within these error groups. The markers مافين (lack of energy) and نفسيتي (psychological distress) were absent (0.0%) from all FN instances, while they appeared in 9.0% and 4.5% of FP instances, respectively. خمول (lethargy) was present in 5.2% of FNs and 19.4% of FPs. Among the FN instances, the highest prevalence was observed for فيني (5.2%) and خمول (5.2%), while all other top markers were present in fewer than 5% of FNs. In FP instances, the highest prevalence was observed for خمول (19.4%), followed by ابي (13.4%) and تبلد (10.4%).

**Table 4:** Systematic error analysis: prevalence of top SHAP markers in false negatives and false positives.

Word	% in False negatives (*N* = 58)	% in False positives (*N* = 67)
مافيني	**0.0**	9.0
نفسيتي	**0.0**	4.5
خمول	5.2	19.4
ضيقه	**0.0**	6.0
طاقهمافيني	**0.0**	6.0
تعبانه	**0.0**	4.5
تبلد	1.7	10.4
مالي	3.4	9.0
فيني	5.2	3.0
ابي	3.4	13.4

Percentages are rounded to one decimal place for readability. Bold values indicate the highest prevalence of a given marker within each error group

## 4 Discussion

This study set out to develop an explainable machine learning framework for depression detection in Arabic tweets, combining a Random Forest classifier with SHAP-based interpretability. The primary aim was to surface clinically meaningful linguistic markers that could bridge the gap between predictive performance and clinical trust, rather than to maximize accuracy. The results demonstrate that this aim has been largely achieved. The RF + SMOTE model achieved acceptable discriminative ability (AUC = 0.80, weighted F1 = 0.79), and the SHAP analysis revealed a coherent set of Arabic depression markers that align closely with established psychiatric symptomatology. Local explanations not only validated correct predictions but also diagnosed the model's primary failure mode, false negatives arising from the absence of prototypical depressive vocabulary. It is important to emphasize that the proposed model is intended solely for research and screening support, and not as a standalone clinical diagnostic instrument. Given the moderate recall for the depressed class and the potential consequences of both false positives and false negatives, any real-world deployment should involve human oversight and should not replace professional clinical judgment.

The choice of Random Forest coupled with SHAP was deliberate and driven by the need for both predictive reliability and theoretical exactness in explanations. Unlike deep neural networks, where computing exact Shapley values is computationally prohibitive and requires approximations that can be misleading, tree-based models allow for exact computation via the TreeSHAP algorithm. This guarantees that the attributions are locally accurate and consistent, meaning that if the model's reliance on a feature increase, its SHAP value never decreases, a theoretical guarantee not offered by other *post-hoc* methods like LIME. This rigorous foundation is critical when the goal is not merely to achieve high accuracy, but to derive clinically trustworthy and verifiable linguistic markers.

### 4.1 Interpretation of model performance and comparison with prior work

The performance of the proposed RF + SMOTE model must be understood within the broader landscape of Arabic mental health NLP. When trained without resampling, the initial RF suffered from a severe class-imbalance problem, achieving a depressed-class recall of only 0.38 and an F1 of 0.47, a pattern consistent with the well-documented challenge of imbalanced mental health datasets (Aljameel et al., 2026). The application of SMOTE improved depressed recall to 0.57 and F1 to 0.53, while maintaining a stable overall accuracy of 0.79 and a weighted F1 of 0.79. The 5-fold cross-validation results (depressed recall = 0.592 ± 0.046, F1 = 0.565 ± 0.037, AUC = 0.823 ± 0.020) confirm that these metrics are stable and not artifacts of a particular data split. The baseline Logistic Regression, with a depressed recall of 0.62 and F1 of 0.54, slightly outperformed RF + SMOTE on the minority class. However, the ensemble method offered a better balance between precision and recall (precision of 0.50 vs. 0.47 for LR), and, critically, its tree-based architecture enables exact TreeSHAP computations, a prerequisite for the primary interpretability aims of this study.

The predictive performance of RF + SMOTE is significantly lower than that reported for deep transformer-based Arabic depression classifiers. For example, accuracies of 97.0% with AraBERT and 97.7% with MARBERT have been reported by Al-Atwi et al. (2025); similarly, accuracy of 93.03% for binary classification and 97.8% for multi-class classification with AraBERT and Multilingual BERT was achieved by Rabie et al. (2025); and Abdulsalam et al. (2024) attained 91% accuracy and an F1 of 0.88 for Arabic suicidality detection using AraBERT (Rabie et al., 2025; Al-Atwi et al., 2025). However, direct numerical comparison with these studies is methodologically unsound because they employed different datasets, tasks, and evaluation protocols. More fundamentally, none of these high-performing models provides interpretability into their decision-making processes; they are effectively black boxes. The deliberate choice of a traditional, transparent architecture reflects a recognized trade-off between marginal performance gains and analytical clarity in explainable AI research (Lundberg and Lee, 2017). A recent meta-analysis of explainable depression detection studies found that the use of SHAP was associated with superior methodological rigor, and that combining SHAP with complementary techniques like LIME enhanced both explainability and reported performance (Trelles et al., 2025).

### 4.2 Comparison of interpretability: SHAP vs. logistic regression coefficients

While Logistic Regression coefficients offer a straightforward global interpretation indicating whether a word generally increases or decreases the predicted probability of depression they are inherently limited to linear, average effects. For example, the LR coefficient for a word like ابي represents its mean influence across all tweets, masking the substantial context-dependent variability revealed by SHAP. The SHAP dependence plot for ابي showed both positive and negative contributions depending on the surrounding text, illustrating how the Random Forest captures non-linear interactions that a linear model cannot represent. Furthermore, LR provides only population-level trends, whereas SHAP generates instance-level explanations, allowing clinicians to understand exactly why a specific tweet was classified as depressed. This granularity is essential for building trust in automated screening tools and for diagnosing model errors such as the false negative case where the absence of high-impact markers like مافيني was the deciding factor. Thus, while LR serves as a valuable baseline for performance comparison, SHAP explanations from tree-based models deliver a richer, more clinically actionable interpretability.

### 4.3 Clinical validity of SHAP-identified markers

The SHAP global importance rankings provide strong evidence that the model has learned clinically valid associations. The five most influential features [مافيني (lack of energy), مافيني (my psyche), خمول (lethargy), ضيقه (tired), and مافيني طاقه (emotional numbness)] show conceptual overlap with DSM-5 symptom domains of major depressive disorder: energy depletion, psychological distress, and anhedonia (American Psychiatric Association, 2013). This convergence between data-driven feature importance and established clinical criteria is a powerful validation of the model's learned representations. In a study applying XGBoost with SHAP to predict depression from sociodemographic and clinical survey data, fatigue and psychological distress similarly emerged as dominant predictors (Mimikou et al., 2025), reinforcing the cross-contextual importance of these features. It should be noted, however, that this alignment is theoretical in nature; formal clinical validation through prospective studies and expert review of the identified markers remains necessary future work.

The dependence plots revealed a consistent monotonic dose-response relationship for all top features, with low vertical dispersion indicating robust effects largely independent of interactions with other terms. No instance with a high TF-IDF value of these words fell into the negative SHAP region, ruling out sign reversals that would suggest artifactual learning. The low activation threshold for تعبانه (producing a positive SHAP shift of approximately +0.02 even at minimal TF-IDF values) is particularly noteworthy. It suggests that the model is highly sensitive to fatigue-related expressions, which aligns with the somatization tendencies documented in Arab populations (Kirmayer, 2001). The presence of multi-word expressions such as طاقة مافيني (rank 9) confirms that the (Aljameel et al., 2026; Friedrich, 2017) n-gram configuration successfully captured collocations that carry psychological meaning beyond their constituent unigrams.

The waterfall plots further illustrated the model's decision logic. In the correctly classified depressed instance, the word انخذلت alone contributed +0.33, overwhelming a modest negative contribution from سعادة (−0.05). In the false-negative case, the absence of prototypical depressive markers such as مافيني and انخذلت, combined with a −0.12 contribution from متحصله, kept the predicted probability at 0.15. This diagnostic capability (revealing not only that the model failed but why) is a distinct advantage of SHAP-based interpretability and directly addresses the transparency deficit in prior Arabic depression detection work.

### 4.4 Linguistic and cultural considerations

The absence of الحمدالله from the vocabulary, because its root الله was removed as a stop word, highlights a broader methodological tension in Arabic mental health NLP. Religious expressions are pervasive in Arabic discourse and often function as coping markers or resignation idioms. The presence of يارب (rank 16) among the top features, despite the removal of many religious terms, suggests that these markers carry a predictive signal even when some are filtered out. This tension has been noted in a recent scoping review of Arabic NLP for mental health, which observed that culturally grounded expressions (particularly religious ones) are frequently lost in standard preprocessing pipelines (Alasmari, 2025). Future work should consider more nuanced stop word strategies that preserve culturally meaningful markers while still filtering genuine noise.

### 4.5 Limitations

Several limitations must be acknowledged. First, despite SMOTE improving depressed recall to 0.57, the model still misses over 40% of genuinely depressive tweets. This limits standalone clinical deployment but is acceptable for an interpretability-focused study where the model serves primarily as a vehicle for SHAP analysis. Second, the AMG dataset is sourced exclusively from Saudi Arabia and may not generalize to other Arabic dialects (Aljameel et al., 2026; Habash, 2010). Third, excluding emojis, punctuation, and non-Arabic characters, while methodologically standard, may have discarded paralinguistic signals that carry emotional information in informal social media discourse. Fourth, the absence of clinical validation through expert review of the SHAP explanations means that the clinical relevance of the identified markers, while theoretically grounded, awaits empirical confirmation.

Fifth, applying SMOTE to high-dimensional, sparse TF-IDF feature spaces may generate synthetic samples that do not correspond to realistic text representations; although this approach is common in NLP pipelines, its effect on downstream SHAP attributions warrants further investigation. Sixth, while we compared six pipelines including SVM and XGBoost, transformer-based models such as AraBERT or MARBERT were not evaluated; future work should explore whether deep learning architectures yield different linguistic markers. Seventh, while we externally validated our model on the Nassar corpus, evaluating the framework on additional Arabic depression datasets such as AraDepSu or AraStress would further strengthen claims of generalizability. Eighth, the AMG dataset does not include any user or author identifiers. As a result, we could not perform user-level splitting or grouped cross-validation, and the data split was performed at the tweet level only. Consequently, multiple tweets from the same individual may appear in both training and test partitions, potentially inflating performance estimates through unintentional user-level leakage. This limitation should be explicitly considered when interpreting the reported metrics, and future studies using datasets with user-level metadata are needed to assess its actual impact. Ninth, some highly ranked SHAP features (e.g., تعاسه, اكتئاب) are direct translations of clinical depression terminology. Their presence may reflect a form of label leakage, where the model capitalizes on explicit diagnostic vocabulary rather than subtle, context-dependent cues. Future work should assess model performance after systematically removing such transparent markers from the feature space.

### 4.6 Implications and future directions

The findings of this study carry implications for both the Arabic NLP and digital mental health communities. The demonstration that a relatively simple, transparent model can surface clinically meaningful markers offers a replicable methodological template for other under-resourced languages. The identified vocabulary- energy depletion, psychological distress, and somatic complaints- could inform the development of culturally sensitive screening instruments or educational resources for mental health literacy campaigns in Arabic-speaking communities.

Several directions for future research emerge. Addressing the class imbalance through alternative strategies such as cost-sensitive learning or generative data augmentation could further improve depressed recall for depression; while we compared ROS and SMOTE in this study, other approaches such as cost-sensitive boosting or GAN-based augmentation warrant further investigation. Additionally, data augmentation with generative models represents a promising direction for creating more realistic synthetic minority samples. Extending the SHAP analysis to transformer-based architectures such as AraBERT and MARBERT would illuminate whether deep models learn similar or fundamentally different linguistic markers, and whether the interpretability–accuracy trade-off can be narrowed. Collecting multi-dialectal Arabic corpora spanning Gulf, Levantine, Egyptian, and Maghrebi varieties would enable cross-dialectal validation. Prospective clinical validation involving mental health professionals would strengthen translational impact. Finally, longitudinal studies tracking individual users over time could move from static classification to dynamic risk monitoring.

## 5 Conclusion

In summary, this study demonstrates that an explainable AI framework (built on a Random Forest classifier augmented with SMOTE and interpreted through SHAP) can surface clinically coherent and culturally grounded Arabic depression markers. The SHAP analysis not only identified which words matter, but also how they combine in individual predictions and why the model fails when it does. This transparency is essential if NLP-based screening tools are to earn a place in real-world mental health care as assistive research instruments, rather than as autonomous diagnostic systems. While performance limitations preclude immediate deployment, the methodological template established here offers a replicable foundation for future research at the intersection of Arabic NLP, explainable AI, and digital mental health. The systematic error analysis further revealed that the model's primary failure mode, false negatives, was consistently associated with the absence of these prototypical linguistic markers, confirming that the model's decisions are grounded in clinically interpretable features.

## Data Availability

Publicly available datasets were analyzed in this study. This data can be found at: https://github.com/LamiaAlhussan/ArabMindGuardDataset/tree/main/Datasets.

## References

[B1] Abdulsalam A. Alhothali A. Al-Ghamdi S. (2024). Detecting suicidality in Arabic tweets using machine learning and deep learning techniques. Arab. J. Sci. Eng. 49, 12729–12742. doi: 10.1007/s13369-024-08767-3

[B2] Alaskar H. Ykhlef M. (2022). Machine learning for detecting depression in Arabic tweets. Int. J. Adv. Comput. Sci. Appl. 13, 345–352. https://www.semanticscholar.org/paper/Depression-Detection-from-Arabic-Tweets-Using-Alaskar/ (Accessed September 28, 2026).

[B3] Alasmari A. A. (2025). scoping review of Arabic natural language processing for mental health. Healthcare 13:963. doi: 10.3390/healthcare13090963 PMC12071875 40361742

[B4] Al-Atwi H. S. Al-Qarni S. M. Al-Marwani W. B. Al-Wabisi A. M. Al-Atwi G. S. (eds.) (2025). “X as a mirror for mental health: the role of AI in analyzing tweets for depression,” in 2025 4th International Conference on Computing and Information Technology (ICCIT) University of Tabuk, (Tabuk: IEEE). doi: 10.1109/ICCIT63348.2025.10989433

[B5] Alhomaid T. A. Alburaidi O. Garallah N. (2025). Prevalence of depression among urban and rural residents of Saudi Arabia compared with other Gulf Cooperation Council countries: a systematic review. Cureus 17:e90321. doi: 10.7759/cureus.90321 PMC12440573 40964597

[B6] Aljameel S. Alqahtani A. Alharbi M. (2026). Machine learning methods for detecting depression in Arabic tweets: a comprehensive performance analysis with enhanced evaluation metrics. Discov. Appl. Sci. 6:120. doi: 10.1007/s44163-026-00842-y

[B7] Almouzini S. Alageel A. (2019). Detecting Arabic depressed users from Twitter data. Procedia Comput. Sci. 163, 257–265. doi: 10.1016/j.procs.2019.12.107

[B8] Al-Musallam N. Al-Abdullatif M. (eds.) (2022). “Depression detection through identifying depressive Arabic tweets from Saudi Arabia: machine learning approach,” in 2022 Fifth National Conference of Saudi Computers Colleges (NCCC), (Makkah: IEEE). doi: 10.1109/NCCC57165.2022.10067346

[B9] AlSagri H. S. Ykhlef M. (2020). Machine learning-based approach for depression detection in twitter using content and activity features. IEICE Trans. Inf. Syst. 103, 1825–1832. doi: 10.1587/transinf.2020EDP7023

[B10] Alzahrani A. (2024). Prevalence of depression and anxiety among university students in Jeddah, Saudi Arabia: exploring sociodemographic and associated factors. Front. Public Health 12:1441695. doi: 10.3389/fpubh.2024.1441695 PMC11670204 39726655

[B11] American Psychiatric Association (2013). Diagnostic and Statistical Manual of Mental Disorders, 5th Edn. Washington, DC: American Psychiatric Publishing. doi: 10.1176/appi.books.9780890425596

[B12] Daoud M. Farraj A. Sultan H. (2025). How many people die by suicide each year? Not 727,000: a systematic review and meta-analysis of suicide underreporting across 71 countries over 122 years. Front. Psychiatry 16:1609580. doi: 10.3389/fpsyt.2025.1609580 PMC12378953 40873674

[B13] Elmajali S. Ahmad I. (2024). Toward early detection of depression: detecting depression symptoms in Arabic tweets using pretrained transformers. IEEE Access 12, 88134–88145. doi: 10.1109/ACCESS.2024.3417821

[B14] El-Ramly M. Abu-Elyazid H. Mo'men Y. Alshaer G. Adib N. Eldeen K. A. El-Shazly M. (eds.) (2021). “CairoDep: detecting depression in Arabic posts using BERT transformers,” in 2021 Tenth International Conference on Intelligent Computing and Information Systems (ICICIS), (Cairo: IEEE). doi: 10.1109/ICICIS52592.2021.9694178

[B15] Friedrich M. J. (2017). Depression is the leading cause of disability around the world. JAMA 317:1517. doi: 10.1001/jama.2017.3826 28418490

[B16] Habash N. Y. (2010). Introduction to Arabic Natural Language Processing. San Rafael, CA: Morgan and Claypool Publishers. doi: 10.1007/978-3-031-02139-8

[B17] Hasan M. R. Islam M. S. Aziz M. A. (2025). Enhanced suicidal ideation detection from social media using a CNN-BiLSTM hybrid model with SHAP-based interpretability. arXiv.

[B18] Hassib M. Hossam N. Sameh J. Torki M. (eds.) (2022). “Aradepsu: detecting depression and suicidal ideation in Arabic tweets using transformers,” in Proceedings of the Seventh Arabic Natural Language Processing Workshop (WANLP), (Abu Dhabi). doi: 10.18653/v1/2022.wanlp-1.28

[B19] Helmy A. Nassar R. Ramdan N. (2024). Depression detection for twitter users using sentiment analysis in English and Arabic tweets. Artif. Intell. Med. 147:102716. doi: 10.1016/j.artmed.2023.102716 38184345

[B20] Kirmayer L. J. (2001). Cultural variations in the clinical presentation of depression and anxiety: implications for diagnosis and treatment. J. Clin. Psychiatry 62(Suppl. 13), 22–28. https://pubmed.ncbi.nlm.nih.gov/11434415/ (Accessed September 28, 2026). 11434415

[B21] Lundberg S. M. Erion G. Chen H. DeGrave A. Prutkin J. M. Nair B. . (2020). From local explanations to global understanding with explainable AI for trees. Nat. Mach. Intell. 2, 56–67. doi: 10.1038/s42256-019-0138-9 PMC7326367 32607472

[B22] Lundberg S. M. Lee S.-I. (eds.) (2017). “A unified approach to interpreting model predictions,” in Advances in Neural Information Processing Systems 30 (NeurIPS) (Long Beach, CA: Curran Associates).

[B23] Maghraby A. Alqahtani M. Alharthi R. (2022). Modern Standard Arabic mood changing and depression dataset. Data Brief 40:107812. doi: 10.1016/j.dib.2022.107999 PMC8902608 35274028

[B24] Malhotra A. Jindal R. (2023). XAI Transformer based approach for interpreting depressed and suicidal user behavior on online social networks. Cogn. Syst. Res. 83:101186. doi: 10.1016/j.cogsys.2023.101186

[B25] Mimikou C. Kokkotis C. Tsiptsios D. Tsamakis K. Savvidou S. (2025). Explainable machine learning in the prediction of depression. Diagnostics 15:1412. doi: 10.3390/diagnostics15111412 PMC12154222 40506984

[B26] Musleh D. Alqahtani A. Alharbi M. (2022). Detecting depression in Arabic tweets using machine learning and NLP techniques. Int. J. Comput. Sci. Netw. Secur. 22, 237–244. doi: 10.32604/cmc.2022.022508

[B27] Rabie E. M. Hashem A. F. Alsheref F. K. (2025). Recognition model for major depressive disorder in Arabic user-generated content. Beni Suef. Univ. J. Basic Appl. Sci. 14:7. doi: 10.1186/s43088-024-00592-9

[B28] Reece A. G. Reagan A. J. Lix K. L. M. Dodds P. S. Danforth C. M. Langer E. J. . (2017). Forecasting the onset and course of mental illness with Twitter data. Sci. Rep. 7:13006. doi: 10.1038/s41598-017-12961-9 29021528 PMC5636873

[B29] Tanaka R. Kodaka M. Fukazawa Y. (2025). Generative contextual insights for social media mental health detection with Shapley Additive Explanations (SHAP)-based interpretation. Web Intell. 23, 543–557. doi: 10.1177/24056456251385719

[B30] Trelles A. Fontaines-Ruíz T. Ponce Rojo A. (2025). Systematic review and meta-analysis of explainable machine learning models for clinical depression detection. Behav. Sci. 15:1476. doi: 10.3390/bs15111476 PMC12649417 41301278

[B31] Zaghouani W. Shlkamy E. S. Bessghaier M. (eds.) (2026). “From posts to pressure: an Arabic dataset about stress and mental-health monitoring,” in Proceedings of the 2nd Workshop on NLP for Languages Using Arabic Script, (Rabat). doi: 10.18653/v1/2026.abjadnlp-1.50

